# Excess mortality from gastric cancer in Korea during the COVID-19 pandemic, 2020–2023

**DOI:** 10.4178/epih.e2026021

**Published:** 2026-05-26

**Authors:** Gyeongil Kim, Ji-Yeon Shin, Yu-Mi Lee, Duk-Hee Lee

**Affiliations:** 1 Department of Epidemiology and Health Promotion, Graduate School of Public Health, Kyungpook National University, Daegu, Korea; 2 Department of Preventive Medicine, Kyungpook National University School of Medicine, Daegu, Korea

**Keywords:** COVID-19, Excess mortality, Gastric cancer, Screening, Survival

## Abstract

The coronavirus disease 2019 (COVID-19) pandemic substantially disrupted cancer care, leading to declines in screening, diagnosis, and treatment. Gastric cancer is particularly vulnerable to such disruptions because reductions in mortality depend largely on early detection and timely treatment. This study estimated excess mortality from gastric cancer in Korea during the COVID-19 pandemic. Expected gastric cancer deaths during the pandemic were estimated by applying linear regression to age-specific mortality trends from 2015 to 2019. Excess mortality was quantified using annual P-scores. Gastric cancer mortality declined steadily before the pandemic, but this decline attenuated during the pandemic period. Annual P-scores increased progressively, from +3.8% in 2020 and +5.5% in 2021 to +10.6% in 2022 and +20.7% in 2023. By age group, individuals aged 60–79 years showed the largest increases, reaching +46.6% in 2023, whereas minimal excess mortality was observed among those aged 20–59 years. Among adults aged ≥80 years, excess mortality became apparent only in 2023 (+12.8%). Overall, a progressive increase in excess mortality due to gastric cancer was observed in Korea during the COVID-19 pandemic, particularly among individuals aged 60–79 years. These findings suggest that the pandemic adversely affected gastric cancer control and underscore the need for continued monitoring.

## GRAPHICAL ABSTRACT

[Fig f3-epih-48-e2026021]

## Key Message

• The COVID-19 pandemic disrupted cancer diagnosis and treatment, particularly for gastric cancer, where early diagnosis and timely treatment are critical for survival.

• Using age-specific gastric cancer mortality trends from 2015–2019, we estimated expected deaths during 2020–2023 and quantified excess mortality in Korea using P-scores.

• The pre-pandemic decline in age-standardized gastric cancer mortality slowed during the pandemic. Excess mortality increased from 3.8% in 2020 to 20.7% in 2023, with the largest increases observed among adults aged 60–79 years.

## INTRODUCTION

The coronavirus disease 2019 (COVID-19) pandemic caused substantial disruptions in medical care for patients with cancer, including declines in screening, diagnosis, treatment, and outpatient visits [1,2]. These disruptions raised concerns about delayed detection and suboptimal cancer management, which may have led to more advanced stages at diagnosis and lower survival rates [3,4]. The magnitude of these effects is expected to vary across cancer types, with the greatest negative impact likely occurring in cancers for which timely detection is central to improving outcomes.

Gastric cancer, which ranks fifth globally in incidence and fourth in mortality [5], is particularly vulnerable to pandemic-related healthcare interruptions because long-term declines in gastric cancer mortality have been driven largely by widespread screening and timely treatment [6]. In East Asian countries with established national screening programs, such as Korea and Japan, screening participation declined during the pandemic [7-9], accompanied by an increase in late-stage diagnoses [10,11]. Similar patterns have been reported in many other countries [12].

Excess mortality provides a comprehensive measure of the overall impact of the pandemic by capturing deaths beyond the number expected under normal circumstances [13]. Although prior research has focused primarily on all-cause mortality, disease-specific excess mortality analyses are essential for identifying differential effects across conditions. For cancer, site-specific excess mortality assessments are particularly important because cancers differ substantially in their natural history, screening pathways, and treatment approaches.

Several studies have compared gastric cancer mortality or survival rates between the pre-pandemic and pandemic periods, but results have varied across countries [14-17]. Because these analyses did not account for long-term secular trends in gastric cancer mortality, which can differ substantially among countries and should be considered in formal excess mortality evaluations, their findings are difficult to interpret. In addition, many studies examined only the early phase of the pandemic when comparing outcomes with those in the pre-pandemic period. However, because the adverse effects of reduced screening and delayed diagnosis often take time to emerge, longer follow-up periods are needed.

Several statistical methods are available for estimating excess mortality. Studies that applied different approaches to estimate all-cause excess mortality in Korea reported broadly consistent results when the models accounted for changes in age structure and long-term mortality trends [18-20]. Importantly, complex statistical models do not necessarily yield valid estimates. For example, a global analysis comparing excess mortality across countries used an ensemble of 6 statistical models [21]. However, this study was widely criticized for producing implausible estimates in many countries because the estimated expected deaths deviated substantially from pre-pandemic trends in observed deaths [22,23]. These findings underscore the importance of graphical comparisons between estimated excess mortality and underlying pre-pandemic mortality trends.

Therefore, this study estimated excess mortality from gastric cancer in Korea from 2020 to 2023 using an age-adjusted linear regression approach previously applied in national analyses of all-cause excess mortality in Korea [19]. This method is intuitive and facilitates comparison with observed pre-pandemic mortality trends. Given the persistent pre-pandemic decline in gastric cancer mortality [24], we hypothesized that this downward trend would attenuate during the pandemic period.

## MATERIALS AND METHODS

### Data and study population

Data on the number of gastric cancer deaths, defined using International Classification of Diseases, 10th revision code C16, from 2015 to 2023 were obtained from Statistics Korea’s cause-of-death database [13]. Data on the registered resident population by 10-year age group, from 0–9 years to ≥90 years, were also obtained from the Korean Statistical Office [13].

### Statistical analysis

Annual crude mortality rates and age-standardized mortality rates for gastric cancer per 100,000 population were first calculated from 2015 to 2023. Age standardization was performed using the 2019 population as the standard population. Excess mortality from gastric cancer was then estimated for each year from 2020 to 2023. Excess mortality was expressed as the P-score, defined as follows: P-score=[(observed deaths−expected deaths)/expected deaths]×100 [14]. Observed deaths corresponded to the recorded number of gastric cancer deaths during 2020–2023, whereas expected deaths were derived from age-specific mortality trends during the pre-pandemic reference period of 2015–2019, stratified by 10-year age group.

Expected deaths were estimated in 3 steps. First, linear regression was applied to age-specific mortality rates from 2015 to 2019 to predict age-specific rates for 2020–2023. Second, the predicted age-specific rates were multiplied by the corresponding age-specific population sizes for each year. Third, the resulting age-specific expected death counts were summed to obtain total expected deaths for each year.

P-scores were calculated for the total population and separately for men and women using the same approach. Age-specific P-scores were also estimated for 3 age groups: 20–59, 60–79, and ≥80 years. These broader age categories were selected to reduce instability caused by small numbers of deaths in narrower age bands. However, because individuals aged ≥40 years are eligible for the national gastric cancer screening program in Korea, additional results are presented after further stratifying the 20–59-year age group at age 40. The 95% confidence intervals (CIs) for P-scores were calculated using the standard normal approximation for Poisson-distributed death counts. Analyses were performed using Microsoft Excel (Microsoft Corp., Redmond, WA, USA) and SPSS version 25.0 (IBM Corp., Armonk, NY, USA).

### Ethics statement

This study used publicly available data and did not require approval from the institutional review board.

## RESULTS

**Figure 1.**
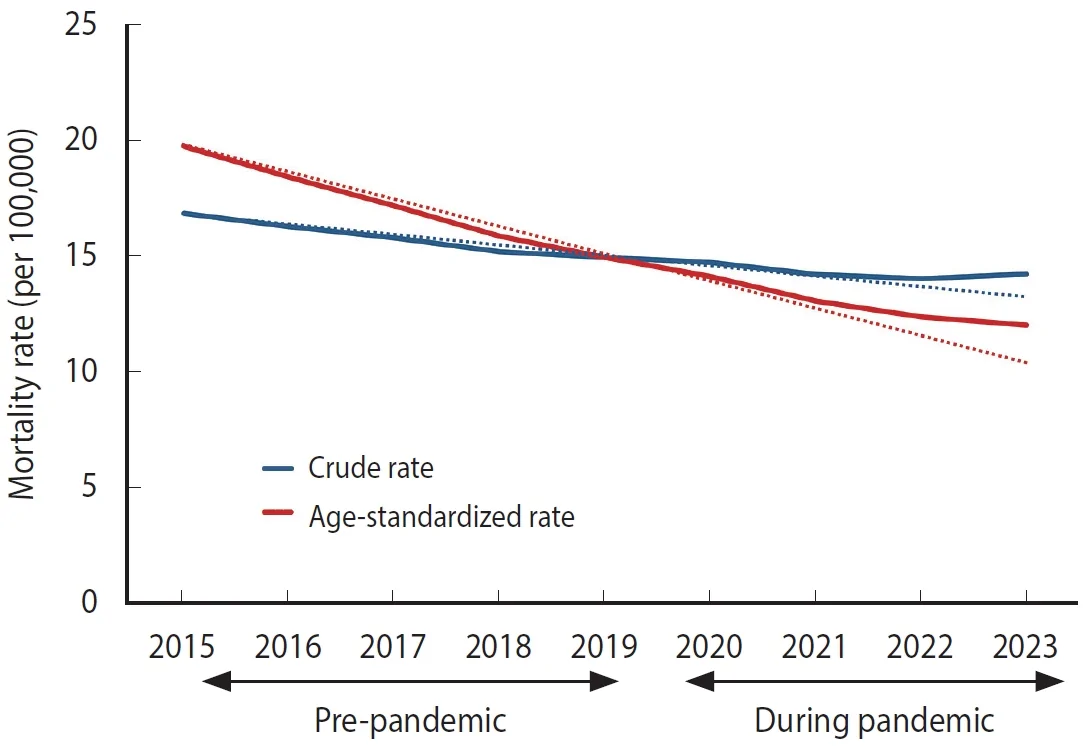
Trends in crude and age-standardized mortality rates for gastric cancer in Korea, 2015–2023. The blue solid line represents the crude mortality rate, and the red solid line represents the age-standardized mortality rate. The dotted lines depict an illustrative extension of the 2015–2019 linear trends into 2020–2023. The 2019 population was used as the standard population for calculating age-standardized mortality.

Figure 1 presents trends in crude and age-standardized mortality rates for gastric cancer from 2015 to 2023. The dotted lines represent a visual extension of the linear downward trends observed during 2015–2019, extended into 2020–2023. These lines provide an illustrative visual guide rather than exact projections derived from the fitted regression equations. Before the pandemic, both crude and age-standardized mortality declined steadily, with a sharper decline in the age-standardized rate, consistent with rapid population aging in Korea. Although mortality continued to decline during the pandemic, the pace of decline progressively attenuated, with this attenuation becoming more pronounced each year.

Table 1 presents observed deaths, expected deaths, and excess mortality, expressed as P-scores, for gastric cancer during the pandemic period. P-scores were +3.8% in 2020, +5.5% in 2021, +10.6% in 2022, and +20.7% in 2023, indicating a gradual and substantial increase in excess mortality over time, with the highest level observed in 2023. These results closely correspond to the visual patterns shown in Figure 1. When stratified by gender, excess mortality was consistently higher among women than among men. Among men, P-scores were +2.2%, +3.7%, +8.8%, and +13.2% from 2020 to 2023, respectively, whereas the corresponding values among women were +8.1%, +5.2%, +14.7%, and +22.7%.

**Figure 2.**
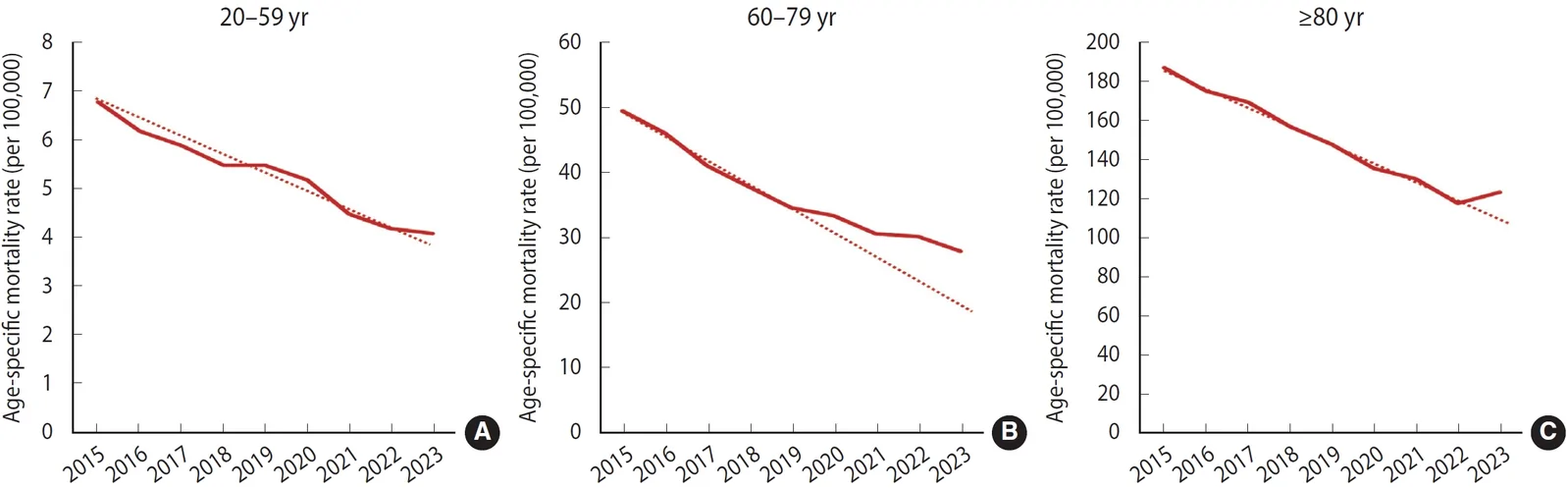
Trends in age-specific gastric cancer mortality rates in Korea, 2015–2023 (A) 20-59 years, (B) 60-79 years, and (C) ≥80 years. The red solid lines represent age-specific mortality rates. The dotted lines depict a visual continuation of the 2015–2019 linear trends into 2020–2023.

Figure 2 presents age-specific gastric cancer mortality trends across 3 age groups. When the pre-pandemic linear trends from 2015 to 2019 were extended into the pandemic period, the 60–79-year age group showed a marked weakening of the previously declining mortality trend. In the ≥80-year group, which had the highest mortality, rates continued to decline through 2022 but shifted upward in 2023. In contrast, no meaningful excess mortality was observed among individuals aged 20–59 years.

Table 2 summarizes observed deaths, expected deaths, and excess mortality by age group. The most pronounced excess mortality occurred among adults aged 60–79 years, with P-scores of +9.7% in 2020, +15.1% in 2021, +32.6% in 2022, and +46.6% in 2023. Among those aged ≥80 years, excess mortality increased only in 2023, reaching +12.8%. Consistent with the visual patterns, no statistically meaningful excess mortality was detected among individuals aged 20–59 years. However, when the 20–59-year age group was further stratified at age 40, P-scores among individuals aged 20–39 years were +33.8%, +38.4%, +92.9%, and +143.4% from 2020 to 2023, respectively, and remained statistically significant despite wide confidence intervals. In contrast, the corresponding values among those aged 40–59 years were +1.2%, −5.6%, −8.3%, and −3.1%.

## DISCUSSION

This study demonstrated that the COVID-19 pandemic adversely affected gastric cancer mortality in Korea. Although gastric cancer mortality continued its long-term decline from 2015 to 2023, the pace of decline weakened during the pandemic period, resulting in measurable excess mortality. Consistent with this pattern, P-scores increased steadily from the first year of the pandemic and continued to rise through 2023.

Age-stratified analyses showed that excess mortality was most pronounced among individuals aged 60–79 years, whereas it remained minimal among those younger than 60 years. Among adults aged ≥80 years, excess mortality was not evident from 2020 to 2022, but a notable increase emerged in 2023. In this oldest age group, the previously declining mortality trend reversed and shifted upward in 2023. When stratified by gender, excess mortality was more evident among women than among men.

Because Korea’s national gastric cancer screening program begins at age 40, the 20–59-year age group was further stratified at age 40. In these analyses, individuals aged 20–39 years exhibited the highest excess mortality, with a steadily increasing trend throughout the pandemic, whereas those aged 40–59 years showed estimates closer to the null or even negative excess mortality. However, findings for the 20–39-year age group should be interpreted with caution because the number of observed deaths was small. Although stratification at age 40 is clinically relevant, interpretations based on broader age groups may be more appropriate in the present study.

To our knowledge, no previous studies have applied formal excess mortality analyses that account for long-term trends in gastric cancer mortality. Existing studies have instead relied largely on comparisons of mortality or survival outcomes between the pre-pandemic and pandemic periods without formal excess mortality methods, yielding heterogeneous results across countries. For example, studies from Japan and Portugal reported decreased survival or increased mortality during the pandemic [14,15], whereas studies from Israel and Spain found no meaningful change in gastric cancer mortality [16,17]. These mixed findings suggest that pandemic-related effects on gastric cancer outcomes differed across countries.

Despite the high incidence of gastric cancer in Korea, widespread screening programs that detect precursor lesions and early-stage tumors have contributed to relatively high 5-year survival rates [25]. Accordingly, disruptions in access to diagnostic and treatment services during the pandemic would be expected to increase excess mortality [9,10]. Although delayed diagnosis and suboptimal treatment both contribute to gastric cancer mortality, their relative influence likely differed across phases of the pandemic. In the early period, such as 2020, disruptions in treatment may have played a greater role in increasing excess mortality. Over time, however, the cumulative effects of delayed screening and postponed diagnostic evaluations would be expected to exert an increasingly substantial impact.

However, a study of Korean adults aged 40–74 years reported a surge in gastric cancer screening, particularly endoscopy, in 2021 after screening rates declined in 2020 [26]. Therefore, delayed diagnosis and suboptimal treatment may not fully explain the progressive increase in excess mortality from 2020 to 2023. Additional factors may also have contributed to excess gastric cancer mortality, although these factors remain unclear.

This study has several limitations. First, although excess mortality was estimated using death certificate data, misclassification of cause of death during the pandemic cannot be ruled out. Previous reports have documented substantial inaccuracies in cause-of-death certification during this period [27]. Such misclassification likely led to underestimation rather than overestimation of gastric cancer mortality because patients with underlying conditions were frequently coded as COVID-19 deaths if they tested positive by polymerase chain reaction. Second, excess mortality estimates can vary depending on the statistical methods used and the choice of reference period. However, prior studies of all-cause excess mortality in Korea have reported relatively consistent results when accounting for age structure and long-term mortality trends [18-20]. Notably, gastric cancer mortality declined steadily from 1999 to 2022, with a steeper decline in earlier years [24]. Consequently, using a longer pre-pandemic reference period would likely produce higher excess mortality estimates than those reported in the present study. Third, excess mortality estimates for subgroups with low gastric cancer mortality may be unstable because of small numbers of deaths. In particular, the markedly elevated excess mortality among individuals aged 20–39 years warrants cautious interpretation. Fourth, although this study included 4 years of data after the onset of the pandemic, the full impact of delayed diagnosis may not yet be evident because these effects can take longer to manifest.

In summary, excess gastric cancer mortality increased steadily from 2020 to 2023, with the greatest impact observed among individuals aged 60–79 years. Given the central importance of early detection and prompt treatment for gastric cancer outcomes, these findings suggest that pandemic-related disruptions adversely affected cancer control efforts. Because the consequences of delayed screening may take years to fully emerge, continued long-term surveillance of mortality trends is essential. Identifying subgroups with disproportionately high excess mortality will also be important for guiding targeted strategies to protect patients with cancer during future public health emergencies.

## Figures and Tables

**Figure f3-epih-48-e2026021:**
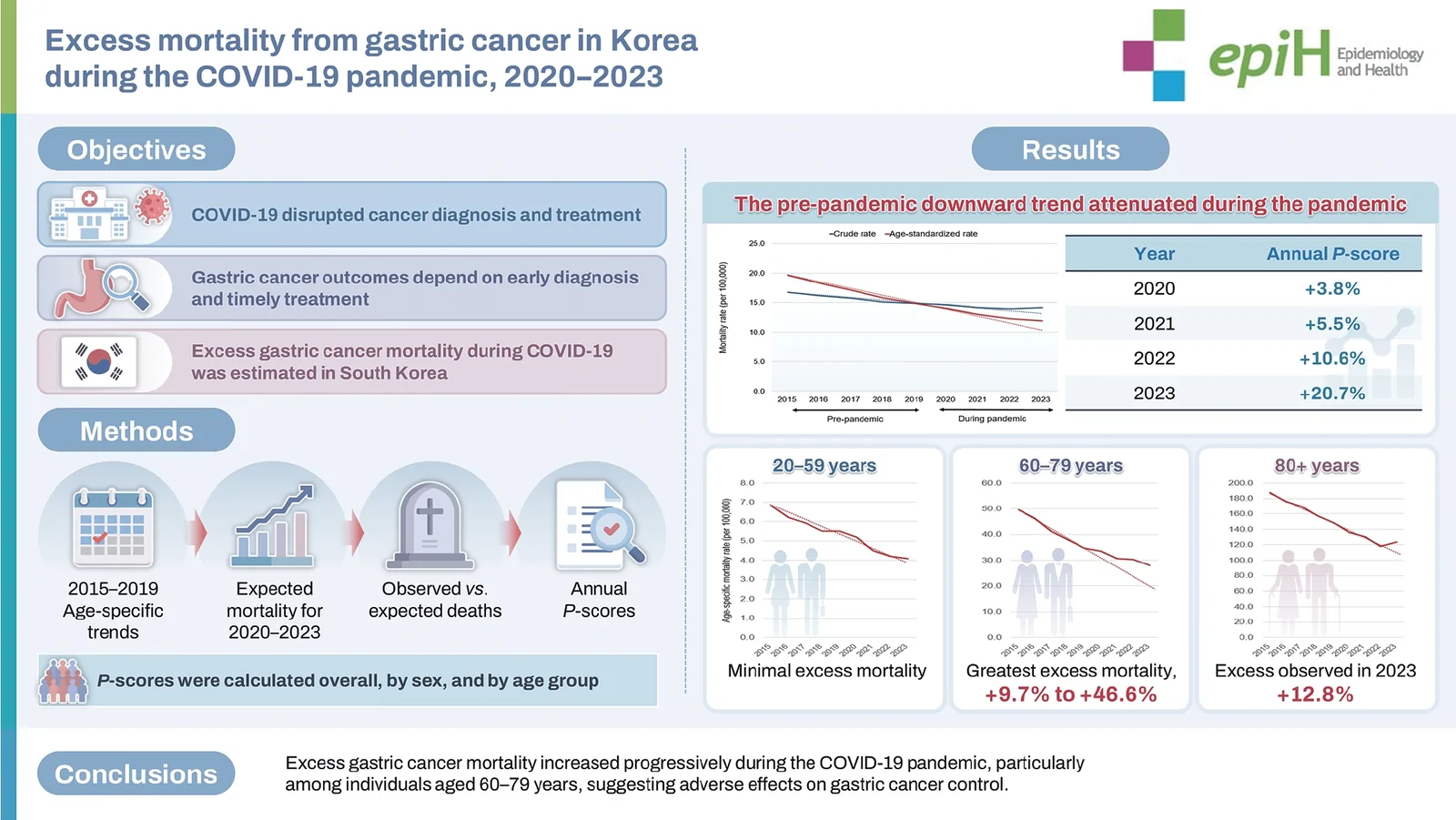


**Table 1.** Excess mortality from gastric cancer in Korea, 2020–2023

Gender	Year	Observed deaths	Expected deaths^1^	P-score (95% CI)^2^
All	2020	7,510	7,235	+3.8 (+1.5, +6.2)
	2021	7,249	6,869	+5.5 (+3.1, +8.0)
	2022	7,147	6,465	+10.6 (+8.0, +13.1)
	2023	7,229	5,989	+20.7 (+17.9, +23.5)
Men	2020	4,955	4,851	+2.2 (-0.7, +5.0)
	2021	4,807	4,634	+3.7 (+0.8, +6.7)
	2022	4,769	4,384	+8.8 (+5.7, +11.9)
	2023	4,620	4,080	+13.2 (+10.0, +16.5)
Women	2020	2,703	2,500	+8.1 (+4.0, +12.2)
	2021	2,480	2,357	+5.2 (+1.1, +9.4)
	2022	2,527	2,203	+14.7 (+10.2, +19.2)
	2023	2,484	2,025	+22.7 (+17.8, +27.5)

CI, confidence interval.

1 Expected deaths were estimated by applying linear regression to age-specific mortality trends from 2015 to 2019 and multiplying the predicted rates by the corresponding population counts for 2020 to 2023.

2 P-scores (%) were calculated as (observed−expected)/expected×100.

**Table 2.** Age-specific excess mortality of gastric cancer in Korea, 2020–2023

Age (yr)	Year	Observed deaths	Expected deaths^1^	P-score (95% CI)^2^
20–59	2020	1,573	1,518	+3.7 (–1.5, +8.8)
	2021	1,365	1,401	–2.7 (–7.9, +2.5)
	2022	1,252	1,288	–2.8 (–8.2, +2.6)
	2023	1,211	1,176	+3.0 (–2.8, +8.8)
60–79	2020	3,361	3,064	+9.7 (+6.0, +13.4)
	2021	3,243	2,817	+15.1 (+11.2, +19.1)
	2022	3,333	2,514	+32.6 (+28.1, +37.1)
	2023	3,176	2,167	+46.6 (+41.5, +51.7)
≥80	2020	2,576	2,629	–2.0 (–5.8, +1.8)
	2021	2,641	2,610	+1.2 (–2.7, +5.1)
	2022	2,562	2,590	–1.1 (–4.9, +2.8)
	2023	2,841	2,519	+12.8 (+8.6, +16.9)

CI, confidence interval.

1 Expected deaths were estimated by applying linear regression to age-specific mortality trends from 2015 to 2019 and multiplying the predicted rates by the corresponding population counts for 2020 to 2023.

2 P-scores (%) were calculated as (observed−expected)/expected×100.

## Data Availability

We used publicly available datasets.
